# Enabling the ActiGraph GT9X Link’s Idle Sleep Mode and Inertial Measurement Unit Settings Directly Impacts Data Acquisition

**DOI:** 10.3390/s23125558

**Published:** 2023-06-14

**Authors:** Hannah J. Coyle-Asbil, Janik Habegger, Michele Oliver, Lori Ann Vallis

**Affiliations:** 1 Department of Human Health and Nutritional Sciences, University of Guelph, Guelph, ON N1G 2W1, Canada; hcoyleas@uoguelph.ca; 2 School of Engineering, University of Guelph, Guelph, ON N1G 2W1, Canada; jhabegge@uoguelph.ca (J.H.); moliver@uoguelph.ca (M.O.)

**Keywords:** accelerometers, ActiGraph, idle sleep mode, low frequency oscillation, robotic motion, wearables

## Abstract

The ActiGraph GT9X has been implemented in clinical trials to track physical activity and sleep. Given recent incidental findings from our laboratory, the overall aim of this study was to notify academic and clinical researchers of the idle sleep mode (ISM) and inertial measurement unit (IMU)’s interaction, as well as their subsequent effect on data acquisition. Investigations were undertaken using a hexapod robot to test the X, Y and Z sensing axes of the accelerometers. Seven GT9X were tested at frequencies ranging from 0.5 to 2 Hz. Testing was performed for three sets of setting parameters: Setting Parameter 1 (ISM_ON_IMU_ON_), Setting Parameter 2 (ISM_OFF_IMU_ON_), Setting Parameter 3 (ISM_ON_IMU_OFF_). The minimum, maximum and range of outputs were compared between the settings and frequencies. Findings indicated that Setting Parameters 1 and 2 were not significantly different, but both were significantly different from Setting Parameter 3. Upon inspection, it was discovered that the ISM was only active during Setting Parameter 3 testing, despite it being enabled in Setting Parameter 1. Researchers should be aware of this when conducting future research using the GT9X.

## 1. Introduction

Accelerometers have been extensively used in research and commercial settings to characterize and evaluate the widespread free-living activity of humans [1], such as sleep, physical activity and sedentary behavior [2] and are being increasingly adopted in clinical research [3]. These devices can elucidate the activity experienced by the wearer by measuring the accelerations experienced by the segment to which they are attached. There is evidence to support the fact that they provide valid measurements across a variety of healthy and clinical populations [3,4,5,6]. Historically, accelerometer data has been widely analyzed in the form of activity counts, and further subdivided into activity intensities using cut-points; however, the considerable disadvantages of these approaches have been highlighted in the literature [7,8]. For example, the algorithms that transform raw accelerometer output into activity counts are often proprietary and have limited the ability to make widespread comparisons [7]. Similarly, the lack of standardization is further perpetuated by the multitude of cut-points that exist [8]. To address this issue, many researchers have recently adopted raw methodological approaches; however, careful consideration of device and software settings is still required to ensure that data collection and processing methods are sound.

In 2014, Actigraph, a popular accelerometer-based manufacturer, released the GT9X Link monitor [9], which has previously been implemented in clinical trials to measure the activity and sleep of clinical populations [4]. Like earlier Actigraph models (e.g., wGT3X-BT), the GT9X monitor houses a triaxial accelerometer that has a dynamic range of ±8 G. This monitor can be worn on the hip, wrist, ankle or thigh. However, in contrast to previous generations of ActiGraph models, the GT9X also contains an inertial measurement unit (IMU), which can be enabled or disabled at initialization. Contained within the IMU there are three sensors: a triaxial magnetometer (dynamic range of ±4800 micro-Tesla), a triaxial gyroscope (dynamic range of ±2000 deg/s) and a secondary triaxial accelerometer (dynamic range of ±16 G). Magnetometers sense directional heading by measuring the strength of the local magnetic field in reference to the Earth’s north pole [10]. Gyroscopes can be used to discern movement related to roll, pitch and yaw by quantifying the rotary rate of a system [11]. As described by ActiGraph, the addition of these sensors allows for more sophisticated free-living positional and movement analyses [9]. It is our understanding that, to date, there are no published free-living assessments that have been performed using the GT9X’s IMU and we suspect this is likely due to the restricted battery life of the device. According to ActiGraph, when the IMU is enabled, the battery life drops to approximately 28 h (https://actigraphcorp.force.com/support/s/article/Link-Battery-Life-and-Memory-Capacity, accessed on 1 September 2022).

The idle sleep mode (ISM) is another setting that users must specify at initialization; ActiGraph has coded this into the firmware of the primary accelerometer to preserve the battery life. When the ISM is enabled, the primary accelerometer will enter a low power state or ‘sleep’ mode after experiencing 10 s of inactivity. Subsequently, the last sampled acceleration value is written into the device’s memory (at the sample rate chosen at initialization) until the monitor detects movement again. In their published online manual, ActiGraph defined this as a ‘fluctuation of less than ±40 Milli-g [12]. The term ‘fluctuation’ can be interpreted in multiple ways; therefore, we contacted ActiGraph representatives who confirmed that the ±40 Milli-g is with respect to a ‘reference’ value. Provided the acceleration stays between −40 Milli-g and +40 Milli-g of the reference value (which would be the last value captured after 10 s of inactivity), the device will enter ISM; to exit ISM, the acceleration must exceed +40 Milli-g or −40 Milli-g from the ‘reference’ value for over 0.1 s. Upon exiting ISM, the timer is reset, and the reference value is changed. To represent this setting more accurately in the current study, we conceptualized the exit from ‘sleep mode’ as an event whereby movement exceeds a threshold band. This feature has been present in ActiGraph models since 2012, but has been seldomly discussed in the literature, and the resulting effects on the data validity have yet to be fully discussed.

As mentioned above, the option of whether to collect IMU data and to enable or disable the ISM in the GT9X is available to researchers. However, while performing unrelated laboratory validation testing, we became aware of an interaction between these two settings, which is not explicitly outlined in software manuals.

The purpose of this observational study is to notify clinical and academic researchers of how the ISM and the IMU interact with one another. It is important to note that the ISM is a software setting designed to impact the primary accelerometer’s data acquisition; in contrast, IMU is a second sensor within the GT9X device that is independent of the primary accelerometer. In the current study, we specifically set out to investigate how the IMU setting combined with the ISM setting affected the data collected by the GT9X primary accelerometer. This was achieved using various combinations of the ISM settings both with and without IMU use in a controlled laboratory setting.

We chose to perform testing in a laboratory environment, as we were able to control the magnitude of acceleration and ensure that our monitors remain in ISM. Moreover, we found in our previous work that the ISM is variable between different devices (ref. [13]; refer to manuscript and it’s corresponding supplementary file); thus, conducting the current study in a controlled laboratory would reduce the inherent variability present in a free-living setting and, in turn, this would allow us to definitively answer our research question.

Given the use instructions provided by ActiGraph, it was hypothesized that the ISM setting and IMU sensor would function separately from one another and that enabling or disabling the IMU would have no significant effect on the ISM in the GT9X primary accelerometer. It should be noted that the IMU sensor has the potential to enhance objective free-living measures (e.g., directional information in addition to the tracking of segment position [14]); thus, understanding the specifics behind these sensors is imperative to draw appropriate behavioral conclusions. In the context of the ISM, this is of particular importance for movement behaviors, such as sleep and sedentary behaviors, that correspond to low acceleration magnitude.

## 2. Materials and Methods

### 2.1. Equipment

For this study, a hexapod robot Mikrolar R-3000 (Mikrolar, Hampton, VA, USA) was used to introduce controlled frequency oscillations. Initially, nine ActiGraph GT9X (*n* = 9) monitors were evaluated; however, during download, communication errors were encountered and data from only seven (*n* = 7) of these monitors were recovered and included. The sampling frequency of the accelerometers was set to 100 Hz and the peak-to-peak displacement of the robot was set to 0.5 cm.

### 2.2. Protocol

**Figure 1:**
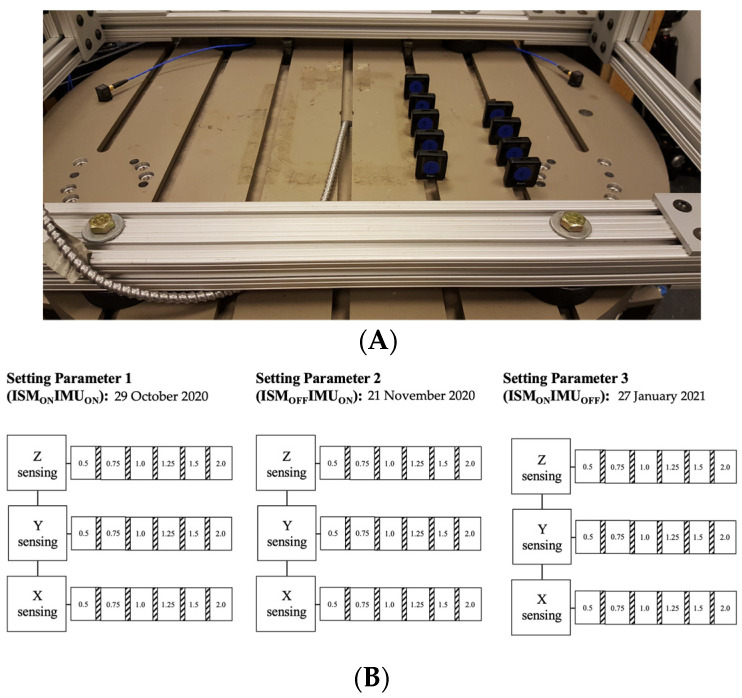
(**A**) A depiction of the experimental setup during the Y sensing axis testing of the GT9X accelerometers, mounted on the hexapod robot; (**B**) dates that the testing of each setting occurred and a schematic depiction of the testing blocks. At each of the six tested frequencies (0.5, 0.75, 1.0, 1.25, 1.5 and 2.0 Hz) three sinusoidal oscillation trials (displacement 0.5 cm; 1 min in duration) were collected; 15 s of no movement between each trial occurred (hatched blocks). A similar testing protocol was used for the X and Z sensing axes.

Highly adhesive tape (https://www.gorillatough.com/product/heavy-duty-mounting-tape/, accessed on 1 September 2022) was used to mount the GT9X monitors to the hexapod robot (Figure 1A). As the GT9X primary accelerometer is a triaxial accelerometer, frequency testing was performed to test the response of all the monitors on the X, Y and Z sensing axes. To achieve this, the orientation of the monitors was altered on the robot for each testing block, whereas the robot was only programmed to perform sinusoidal oscillations along its Z axis. Three separate tests for the initialization setting parameters were performed to evaluate the functionality of the IMU and ISM. The IMU and ISM were enabled for Setting Parameter 1 (ISM_ON_IMU_ON_); the IMU was enabled, and the ISM was disabled for Setting Parameter 2 (ISM_OFF_IMU_ON_); finally, the IMU was disabled, and the ISM was enabled for Setting Parameter 3 (ISM_ON_IMU_OFF_). To start, an unperturbed trial (30 s; used for bias removal) was captured and, subsequently, six low-frequency oscillations were applied by the hexapod. This included: 0.5, 0.75, 1.0, 1.25, 1.5 and 2.0 Hz (Figure 1B). At each frequency, three trials were collected and had a duration of 1 min, with 15 s of rest between each trial. As shown in Figure 1B, these perturbations were consistent across the different sets of settings and sensing axes. To reiterate, the 0.5 cm displacement remained consistent for all of the frequencies tested. Our research group investigates the movement behaviors of pediatric populations and their caregivers. Given this framework, we chose these frequencies for the current study as they are consistent with previously published magnitudes corresponding to low-intensity, free-living behaviors in young children [15,16,17]. The theoretical accelerations were computed in an earlier study from our group using the equation: a = (2πf)2 × d (see Supplementary file of [13]).

### 2.3. Data Analyses

Following data collection, the raw data from the ActiGraph monitors were downloaded using the ActiLife software (ActiGraph, version 6.13.4; Pensacola, FL, USA; firmware version 1.7.2) and exported to timestamped .csv files. A computer code was developed in Python (version 3.7.0, Python Software Foundation, https://www.python.org/, accessed on 1 September 2022) using the SciPy [18], Pandas [19] and NumPy [20] libraries was developed. First, any ambient high-frequency noise that the primary GT9X accelerometer may have collected was removed using a low pass fourth-order Butterworth filter (filter cut-off frequency: 15 Hz). Subsequently, the signal bias resulting from acceleration due to gravity and its orientation on the robot was removed by taking the mean value of the unperturbed trial collected at the start of each testing session and axes and subtracting it from the data collected during each of the oscillation trials to isolate for acceleration due to movement. The outcome variables that were compared included the minimum, maximum and range of the outputs during each of the tested frequencies, and were captured and calculated for comparison purposes. The maximum and minimum values obtained during each measurement period reflect the maximum accelerometer outputs obtained in the positive and negative direction, with respect to how the ActiGraph monitors axes are defined. These measures can be used to provide context and comparisons to studies that have measured the low amplitude movement of humans, such as sedentary and stationary behavior [15,16]. As a composite measure, the range of accelerometer outputs was used to reflect the minimum and maximum values produced for each measurement period. Prior to the calculation of these outcome measures, the first and last 15 s of each trial were excluded to ensure that the robot had reached a steady state and that the data quantified by the accelerometers were reliable. After verifying that no obvious data outliers were present across the three trials (via visual inspection), an aggregated (average across the three trials) value for the maximum, minimum and range of outputs was derived for each device at each frequency, setting and axes.

### 2.4. Statistical Analyses

To compare the effect of setting parameters and frequency, two-way repeated measurement ANOVAs were conducted for the X, Y and Z sensing axes to determine the effect of the ISM and IMU interaction at each frequency. In case Mauchly’s test of sphericity was violated, the Greenhouse-Geisser estimates of sphericity were used to correct for the degrees of freedom. Bonferroni adjustments were used for the pairwise comparisons where appropriate. The statistical analyses were conducted using IBM SPSS (Version 26; IBM Corp, Armonk, NY, USA). A *p*-value of less than 0.05 was considered statistically significant and the necessary assumption tests were performed.

## 3. Results

**Figure 2:**
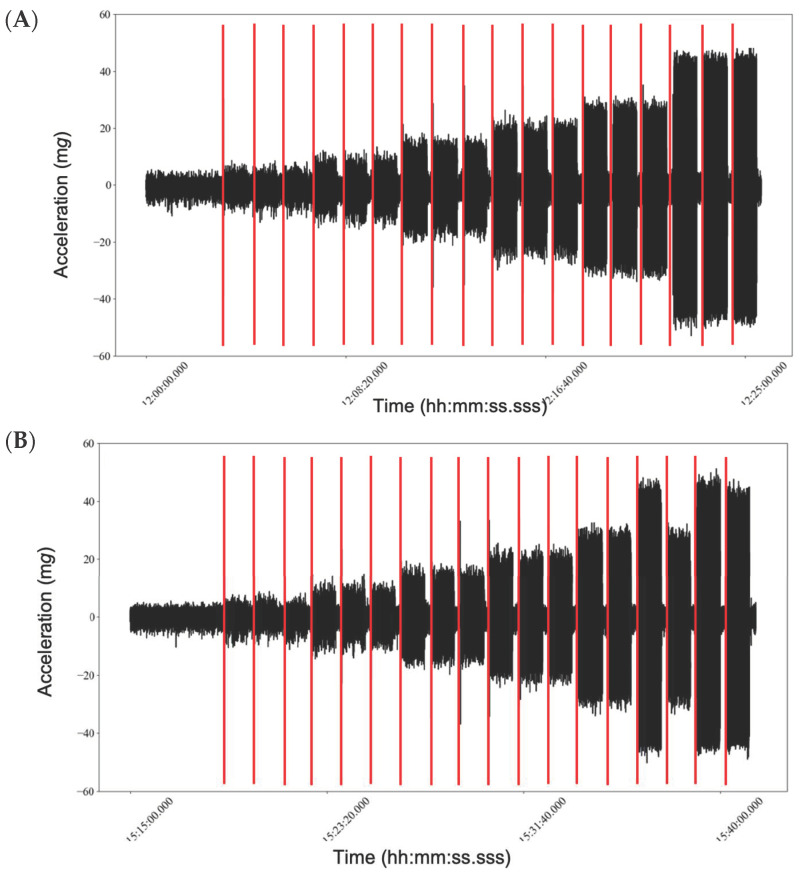

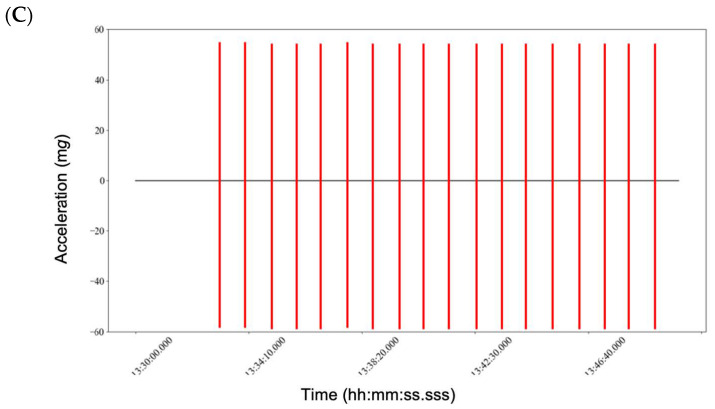
X-axis sensing testing block for Setting Parameter 1 (ISM_ON_IMU_ON_; (**A**)), 2 (ISM_OFF_IMU_ON_; (**B**)) and 3 (ISM_ON_IMU_OFF_; (**C**)). Depicted are three 1-min trials, with 15 s of no movement in between trials (indicated by the red lines) collected at frequencies: 0.5, 0.75, 1.0, 1.25, 1.5 and 2.0 Hz. Note that one of the 1-min trials that took place for the 1.5 Hz testing occurred following the first 2.0 Hz trial for Setting 2.

**Figure 3:**
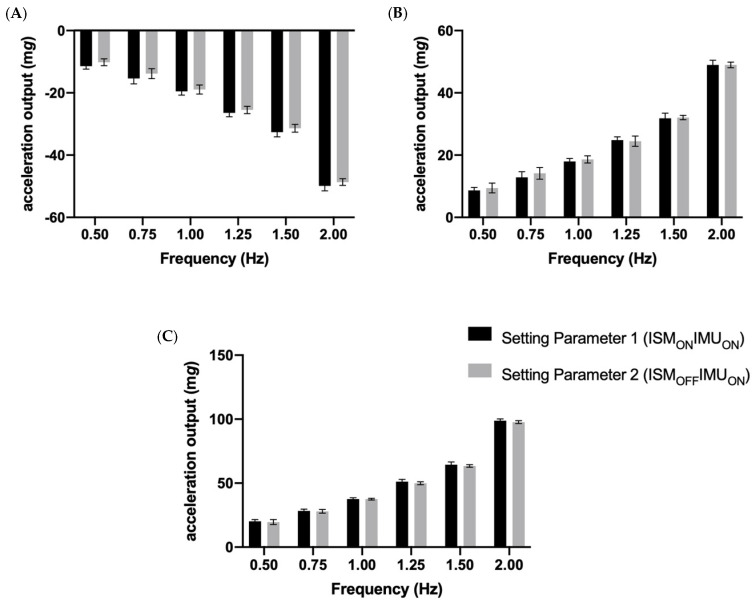
The mean and standard deviation of (**A**) the minimum; (**B**) the maximum; and (**C**) the range output of the GT9X accelerometer during all of the tested frequencies, according to Setting Parameter 1 (ISM_ON_IMU_ON_) and 2 (ISM_OFF_IMU_ON_) during the X sensing testing block.

**Figure 4:**
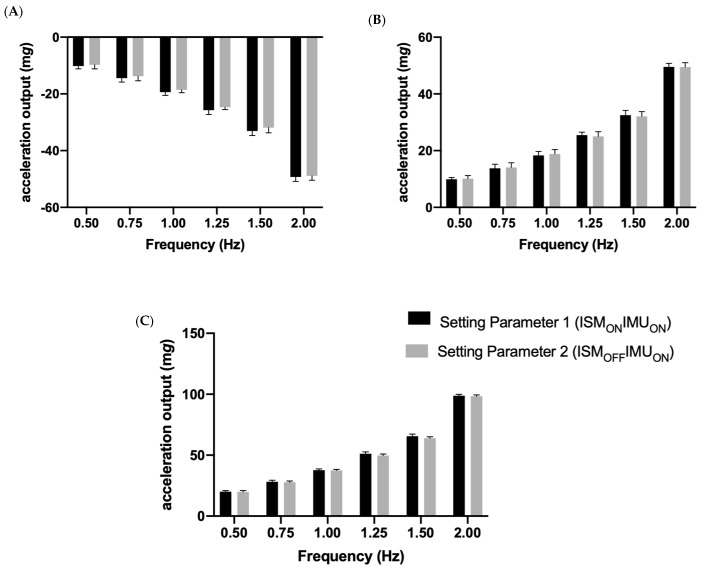
The mean and standard deviation of (**A**) the minimum; (**B**) the maximum; and (**C**) the range output of the GT9X accelerometer during all of the tested frequencies, according to Setting Parameter 1 (ISM_ON_IMU_ON_) and 2 (ISM_OFF_IMU_ON_) during the Y sensing testing block.

**Figure 5:**
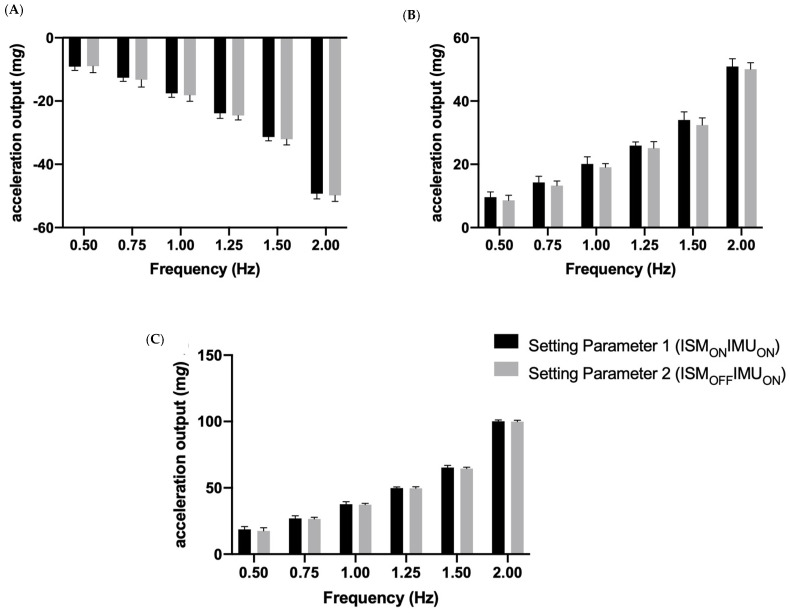
The mean and standard deviation of (**A**) the minimum; (**B**) the maximum; and (**C**) the range output of the GT9X accelerometer during all of the tested frequencies, according to Setting Parameter 1 (ISM_ON_IMU_ON_) and 2 (ISM_OFF_IMU_ON_) during the Z sensing testing block.

When the devices were initialized under Setting Parameter 3 (ISM_ON_IMU_OFF_), they all remained in ISM for the duration of the testing period; i.e., an output of zero (see Figure 2 for representative plots of one device under testing during each of the settings). For this reason, the data obtained by the monitors initialized under Setting Parameter 3 (ISM_ON_IMU_OFF_) were excluded from Figure 3, Figure 4 and Figure 5. Table 1 shows the mean maximum (SD) outputs according to each frequency, setting parameter and axes.

### 3.1. The X Sensing Axes

For the X sensing axis, the two-way repeated measures ANOVA revealed a significant interaction effect between the setting parameter and frequency for all three outcome measures; minimum F(10, 60) = 894.003 (*p* < 0.001), maximum F(10, 60) = 1319.464 (*p* < 0.001) and range F(10, 60) = 2489.988 (*p* < 0.001). Given these findings, the simple effects were further analyzed using a Bonferroni adjustment.

For the simple effect frequency, it was found that the minimum, maximum and range of outputs captured by Setting Parameter 1 (ISM_ON_IMU_ON_) and 2 (ISM_OFF_IMU_ON_), at every tested frequency, were not significantly different from one another (*p* > 0.05; see Figure 3). Setting Parameter 1 (ISM_ON_IMU_ON_) and 2 (ISM_OFF_IMU_ON_) were found, however, to be significantly different from Setting Parameter 3 at all the tested frequencies (ISM_ON_IMU_OFF_; *p* < 0.05). This significant difference was found to increase as the frequency increased.

For the simple effect of the setting parameter, the minimum, maximum and range were significantly different (*p* < 0.05) between all frequencies (0.5, 0.75, 1.0, 1.25, 1.5 and 2.0 Hz) when measured using Setting Parameter 1 (ISM_ON_IMU_ON_; Figure 3). The same findings were found for Setting Parameter 2 (ISM_OFF_IMU_ON_; Figure 3), whereby the minimum, maximum and range were significantly different between all of the tested frequencies (*p* < 0.05). However, for Setting Parameter 3 (ISM_ON_IMU_OFF_), it was found that the minimum, maximum and range were not significantly different (*p* > 0.05) between the different frequencies. 

### 3.2. The Y Sensing Axes

A significant interaction effect between frequency and setting parameter was found for the minimum F(10, 50) = 1029.213, maximum F(10, 50) = 1628.548 and range F(10, 50) = 2537.913, similar to the results from the X axis. Accordingly, the interaction was further explored by analyzing the simple main effects using a Bonferroni adjustment.

Like the simple effect analysis of frequency for the X axis, there were no significant differences between the minimum, maximum and range, measured at the different frequencies, between Setting Parameter 1 (ISM_ON_IMU_ON_) and 2 (ISM_OFF_IMU_ON_; *p* > 0.05). However, the dependent variables captured by Setting Parameter 3 (ISM_ON_IMU_OFF_) were found to be significantly different than these setting parameters (*p* < 0.05) across all of the frequencies, and again, this mean difference increased as the tested frequency increased.

For the simple effect of the setting parameter, the statistical analyses revealed that the minimum, maximum and range of outputs were significantly different (*p* < 0.05) when measured using Setting Parameter 1 (ISM_ON_IMU_ON_) and Setting Parameter 2 (ISM_OFF_IMU_ON_; Figure 4) at all of the tested frequencies. Similar to the X sensing axis, when measured using Setting Parameter 3 (ISM_ON_IMU_OFF_), the dependent variables were not significantly different (*p* > 0.05) across the different frequencies.

### 3.3. The Z Sensing Axes

Similar to the X and Y results, the two-way ANOVA for the Z sensing axis was similar, revealing a significant interaction effect (setting parameter x frequency) for the minimum F(10, 60) = 975.838, maximum F(10, 60) = 824.263 and range F(10, 60) = 1427.463. Subsequently, the data were analyzed for the simple effects of both frequency and setting parameter. This was performed using a Bonferroni adjustment.

For the simple effect of frequency, it was apparent that the minimum, maximum and range were not significantly different between Setting Parameter 1 (ISM_ON_IMU_ON_) and 2 (ISM_OFF_IMU_ON_) at all of the tested frequencies (Figure 5). In contrast, Setting Parameter 3 (ISM_ON_IMU_OFF_) was found to be significantly different than Setting Parameter 1 (ISM_ON_IMU_ON_) and 2 (ISM_OFF_IMU_ON_) across all frequencies with respect to the minimum, maximum and range. These mean differences increased as the frequency increased.

For the simple effect of the setting parameter, it was found that the minimum, maximum and range of outputs were significantly different (*p* < 0.05) between the different frequencies when Setting Parameter 1 (ISM_ON_IMU_ON_) and Setting Parameter 2 (ISM_OFF_IMU_ON_) were tested (Figure 5). Again, similar to the X and Y sensing results, the dependent variables were not significantly different (*p* > 0.05) during the Setting Parameter 3 (ISM_ON_IMU_OFF_) testing set.

## 4. Discussion

This observational study intended to notify researchers using the ActiGraph GT9X accelerometer of how the ISM, a setting coded in the firmware of the primary accelerometer, and the IMU, a sensor independent of the primary accelerometer, interact with one another. This was achieved by comparing the raw acceleration output of the primary accelerometer (minimum, maximum and range) captured during systematically controlled, laboratory-based low-frequency oscillations and different combinations of these setting parameters.

In contrary to our initial hypothesis, the findings indicate that the ISM and IMU do not separately function. Based on how this setting and sensor are described in software manuals and communicated during the initialization process in the ActiLife software (ActiLife v6.13.4), our hypothesis was that the data quantified when the ISM was enabled would not be impacted by enabling or disabling the IMU sensor. The results, however, demonstrate the inverse to be true. Specifically, our results demonstrate that, at all frequencies, the raw output of the primary accelerometer obtained using Setting Parameter 3 (ISM_ON_IMU_OFF_) was significantly different from Setting Parameter 1 (ISM_ON_IMU_ON_) and 2 (ISM_OFF_IMU_ON_). It was initially assumed that the minimum, maximum and range would be equivalent between Setting Parameter 1 and 3, as the ISM was enabled in both. However, the dependent variables were found to not be significantly different between Setting Parameter 1 and 2, which can be attributed to enabling the IMU sensor.

Furthermore, when the ISM was enabled and confirmed to be active via visual inspection (during the testing of Setting Parameter 3), the monitors remained in ‘sleep’ mode for all six of the tested frequencies; as a result, the minimum, maximum and range accelerations measured were not statistically different across the frequencies. In contrast, the findings confirm that the GT9X primary accelerometer was able to differentiate all of the frequencies; i.e., significantly different minimums, maximums and ranges measured at 0.5, 0.75, 1.0, 1.25, 1.5 and 2.0 Hz (see Figure 3, Figure 4 and Figure 5), when testing was performed using Setting Parameter 1 (ISM_ON_IMU_ON_) and 2 (ISM_OFF_IMU_ON_). This can be explained by the fact that, during these settings, the accelerometers never entered ‘sleep mode’. If researchers are unaware of this interaction, it is possible that they may introduce inconsistencies into their data collection. For example, in longitudinal studies, researchers may initialize monitors at baseline with Setting Parameter 3 (ISM_ON_IMU_OFF_) and at follow-up with Setting Parameter 1 (ISM_ON_IMU_ON_) to collect additional IMU data, not knowing that this would affect the data collected by the primary accelerometer. This would be of particular importance for researchers who may use the GT9X to monitor low-intensity behaviors, such as sleep [4,21] and sedentary behavior [5,16], as these activities produce movements with low acceleration magnitudes that may be more affected by the ISM setting choice.

In a recent scoping review investigating accelerometer methods, 480 of the 639 articles reviewed reported using ActiGraph accelerometers (Breau et al., 2022). The GT9X Link accelerometer has been used to track the free-living movement behaviors of healthy adults [22,23] and children [15,16,24], as well as clinical populations [3,4,5] where the potential to improve energy expenditure estimates using the IMU data is currently a focus of different research groups [25,26]. As previously mentioned, our initial curiosity regarding the device settings was sparked by an incidental finding that was discovered during unrelated validation testing on our ActiGraph devices. Accordingly, ActiGraph was contacted to clarify our results and manufacturer representatives were able to confirm how these settings interact. Regardless of user selection, when the IMU is enabled, the ISM is automatically disabled. It was indicated to us that the demand on battery life from the IMU was so great that enabling the ISM complicated the amount of battery life that would have been preserved. The manufacturer also noted that ISM testing is not a consistent part of their quality control protocols. We, like other scientists in this field of study (e.g., [27]), feel that it is important for researchers to understand how decisions made during initialization subsequently affect data acquisition; this is especially critical for the current data collection settings, where findings are not intuitive. Although not specifically tested in this study, we did consider including a fourth Setting Parameter, where the IMU sensor and ISM setting were both disabled (ISM_OFF_IMU_OFF_). The description of these settings by ActiGraph, in addition to correspondence with the company coupled with preliminary findings from this study, indicated that enabling the IMU will automatically disable the ISM. Given that, for Setting Parameter 4, neither functionality is enabled, we would not expect the IMU to collect any data, and therefore, the primary accelerometer would not enter ISM; thus, this combination of setting options was not included in the current analyses.

It is our understanding that we are the first study to report this incidental finding and explore this interaction between the ISM setting and the IMU sensor. As previously expressed [24], the short battery expectancy of the GT9X monitors when the IMU is enabled is likely why very few research groups have not explored the functionality, especially in free-living settings. It is not usual practice for researchers to describe whether the ISM setting parameter was enabled or disabled; even research groups who have been explicit about their selection have not expanded on their processing decisions (e.g., [4,5,21,28,29,30,31]). For example, Arvidsson et al., in 2019, simply stated that they enabled the ISM when they collected physical activity data from children, adolescents and adults; Buchan et al., in 2020, captured the physical activity of adults and reported disabling the ISM, but did not expand on the rationale for this choice. Consequently, how the ISM affects data validity during free-living analyses remains unclear, and the degree of data comparability across studies has not yet been characterized. Obtaining consensus or agreement on device settings across different study protocols may not be practical given the breadth of different research questions addressed using the GT9X device; however, we maintain that consistent methodologies throughout one particular study are essential.

## 5. Conclusions

To conclude, the findings from this observational study describe the relationship between the GT9X’s IMU sensor and ISM setting, and the resulting effect on data acquisition in a controlled laboratory setting. When the IMU is enabled and collecting data in the GT9X, it will automatically disable the ISM in the GT9X primary accelerometer, regardless of its specification at initialization; this finding is in contrast to how it is described in the ActiLife software and user manuals. As such, the GT9X primary accelerometer will not enter sleep mode when motionless. Our recommendation for researchers moving forward is to take particular care when enabling the IMU sensor and remain consistent with their use of these setting parameters, especially for longitudinal research studies. Future research should investigate how the validity of raw data is affected by enabling the ISM versus disabled and determine how the IMU can strengthen motion analyses of free-living human movement.

## Figures and Tables

**Table 1:** Mean maximum (SD) acceleration values corresponding to each axis, frequency and setting parameter.

Axes	Frequency (Hz)	Setting Parameter	Acceleration (Milli-g)
X	0.5	1 (ISM_ON_IMU_ON_)	8.68 (0.97)
		2 (ISM_OFF_IMU_ON_)	9.45 (1.58)
		3 (ISM_ON_IMU_OFF_)	0.00 (0.00)
	0.75	1 (ISM_ON_IMU_ON_)	12.87 (1.83)
		2 (ISM_OFF_IMU_ON_)	14.15 (1.86)
		3 (ISM_ON_IMU_OFF_)	0.00 (0.00)
	1.0	1 (ISM_ON_IMU_ON_)	17.98 (0.96)
		2 (ISM_OFF_IMU_ON_)	18.61 (1.15)
		3 (ISM_ON_IMU_OFF_)	0.00 (0.00)
	1.25	1 (ISM_ON_IMU_ON_)	24.81 (1.07)
		2 (ISM_OFF_IMU_ON_)	24.46 (1.65)
		3 (ISM_ON_IMU_OFF_)	0.00 (0.00)
	1.5	1 (ISM_ON_IMU_ON_)	31.81 (1.65)
		2 (ISM_OFF_IMU_ON_)	32.08 (0.71)
		3 (ISM_ON_IMU_OFF_)	0.00 (0.00)
	2.0	1 (ISM_ON_IMU_ON_)	48.98 (1.48)
		2 (ISM_OFF_IMU_ON_)	48.97 (0.92)
		3 (ISM_ON_IMU_OFF_)	0.00 (0.00)
Y	0.5	1 (ISM_ON_IMU_ON_)	9.92 (0.59)
		2 (ISM_OFF_IMU_ON_)	10.11 (1.12)
		3 (ISM_ON_IMU_OFF_)	0.00 (0.00)
	0.75	1 (ISM_ON_IMU_ON_)	13.8 (1.45)
		2 (ISM_OFF_IMU_ON_)	14.08 (1.64)
		3 (ISM_ON_IMU_OFF_)	0.00 (0.00)
	1.0	1 (ISM_ON_IMU_ON_)	18.32 (1.43)
		2 (ISM_OFF_IMU_ON_)	18.85 (1.52)
		3 (ISM_ON_IMU_OFF_)	0.00 (0.00)
	1.25	1 (ISM_ON_IMU_ON_)	25.46 (1.04)
		2 (ISM_OFF_IMU_ON_)	25.03 (1.63)
		3 (ISM_ON_IMU_OFF_)	0.00 (0.00)
	1.5	1 (ISM_ON_IMU_ON_)	32.53 (1.65)
		2 (ISM_OFF_IMU_ON_)	32.06 (1.73)
		3 (ISM_ON_IMU_OFF_)	0.00 (0.00)
	2.0	1 (ISM_ON_IMU_ON_)	49.54 (1.22)
		2 (ISM_OFF_IMU_ON_)	49.46 (1.55)
		3 (ISM_ON_IMU_OFF_)	0.00 (0.00)
Z	0.5	1 (ISM_ON_IMU_ON_)	9.63 (1.67)
		2 (ISM_OFF_IMU_ON_)	8.62 (1.62)
		3 (ISM_ON_IMU_OFF_)	0.00 (0.00)
	0.75	1 (ISM_ON_IMU_ON_)	14.26 (1.94)
		2 (ISM_OFF_IMU_ON_)	13.3 (1.43)
		3 (ISM_ON_IMU_OFF_)	0.00 (0.00)
	1.0	1 (ISM_ON_IMU_ON_)	20.15 (2.25)
		2 (ISM_OFF_IMU_ON_)	19.06 (1.17)
		3 (ISM_ON_IMU_OFF_)	0.00 (0.00)
	1.25	1 (ISM_ON_IMU_ON_)	25.92 (1.15)
		2 (ISM_OFF_IMU_ON_)	25.14 (2.04)
		3 (ISM_ON_IMU_OFF_)	0.00 (0.00)
	1.5	1 (ISM_ON_IMU_ON_)	34.01 (2.57)
		2 (ISM_OFF_IMU_ON_)	32.45 (2.24)
		3 (ISM_ON_IMU_OFF_)	0.00 (0.00)
	2.0	1 (ISM_ON_IMU_ON_)	50.93 (2.44)
		2 (ISM_OFF_IMU_ON_)	50.07 (2.05)
		3 (ISM_ON_IMU_OFF_)	0.00 (0.00)

## Data Availability

Data available on request.
